# Anemia

**Published:** 1866-08

**Authors:** David Wooster


### SELECTIONS.

*Anemia.—Fibrin coagulates only under the influence of coagulating substance, termed globulin.* By David Wooster, M. D.

A. Schmidt proved by researches and analysis of remarkable exactness, that fibrin does not exist in the blood. It can there be found merely in the condition of a fibrinogenous liquid matter, as Virchow had already suggested. This substance is formed in the parenchymas; it is modified in the lymphatic vessels, then enters the blood where it undergoes a new transformation under the influence of oxygen.

Furthermore, the globules of the blood contain a substance which, certain undetermined circumstances, and always after death, acts as a coagulating principle. It is not as foreign bodies that globules produce coagulation, but by virtue of a substance in their constitution.

This substance is not a gaseous fluid, for oxygen merely hastens coagulation, but is not the cause of it; carbonic acid gas, far from favoring solidification of the clot, diminishes the tendency to coagulation. The coagulating principle not being any gas pertaining to the blood, can only be a liquid material, and this is positively demonstrated by experiment. The preferred location of this coagulating material is in the red globules, and particularly in the hemato-crystalin; if a little defibrinated blood containing red globules be added to a given quantity of chyle, which we know coagulates slowly and with difficulty, on the instant coagulation will take place. Globules then are true exciters of coagulation. This power of inducing coagulation also exists in the cells of the connective tissue, which are in the vascular walls, but in an infinitely less degree; it is also found in the lymph which contains only white globules.

Now, the coagulative power not being peculiar to the red globules, it must pertain to a principle common to all the histologic elements; this substance, according to A. Schmidt, is globulin. *The plastic power of a liquid may be ascertained by the amount of globulin it contains.* In the blood globule, it is an integral part of the globule itself, and this fact explains the superior coagulating energy manifested by these globules.

To obtain this plastic (coagulating) substance, a current of carbonic acid is passed through a very dilute solution of crystals of blood, an amorphous white deposit is thus formed, which, after having been dissolved in a weak alkaline solution, exhibits coagulating properties in the highest degree.

This substance exhibits the same reaction as the globulin of Berzelius, and particularly that which is extracted from crystalin.

Globulin is found with the same properties in all tissues formed by cells; but in the normal condition of life, it (the globulin) could not exercise its peculiar power without coagulating the blood in the whole vascular system; there must be a power in the walls of the vessels themselves of transforming the globulin into coagulable matter, under the influence of the ozone of the blood. Thus, in fact, the slower the blood is drawn from a vein the more slowly it coagulates subsequently. Why? Because contact of the blood with the walls of the living vessel prevents it from coagulation. But, as soon as the irritability of a vessel is lost, it loses property of decomposing globulin, then coagulation takes place easily, by the intervention of hematin, which preserves for a much longer time its coagulating power, to which now nothing is opposed; hence coagulation after death.

Thus, in the living organism, fluidity of the blood is maintained by the vascular walls; but vitality does not act alone; it is hastened by a simple physical action, that is, by the decomposition of the globulin or its transformation into fibrinogen.

Besides, the vital action, that it may be efficient, demands a purely physical condition, which is regularity of the current of the blood; if an obstacle interferes with the circulation, or if an inequality in the walls occurs, by any lesion whatsoever, the wall can no longer be considered as living, that is, in normal life, at that point; at that point it ceases to destroy the globulin, and the blood remains free to exclusive physical action; it coagulates at the diseased point; it stops at the roughened point, and this first coagulum once formed, becomes the focus of successive stratifications.

Schmidt, who described these properties of globulin, stopped at the chemical limit of his investigation, without taking into account the physiological applications here suggested.—*Pacific Medical and Surgical Journal.*

